# Supplementary figures and images for: Starting at the community: Treatment-seeking pathways of children with suspected severe malaria in Uganda
Source: PLOS Glob Public Health. 2023 Jul 5;3(7):e0001949. doi: 10.1371/journal.pgph.0001949 (PMC10321646; doi:10.1371/journal.pgph.0001949)

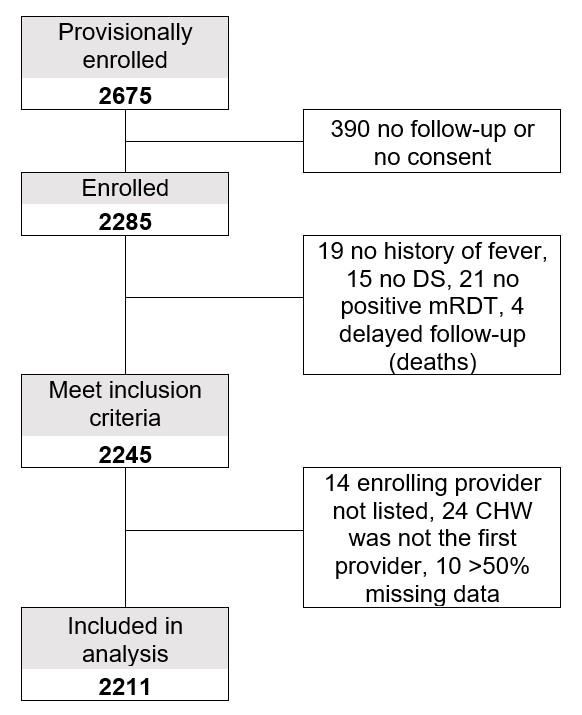

Supplement: S1 Fig — The reasons for study exclusion in the left-hand boxes are not mutually exclusive, i.e. the numbers may add up to a higher total than the number of children actually excluded. DS = danger sign, mRDT = malaria rapid diagnostic test, CHW = community health worker. (TIF) [file pgph.0001949.s005.tif]
